# Noninvasive Mapping System for the Stereotactic Radioablation Treatment of Ventricular Tachycardia: A Case Description

**DOI:** 10.3390/jcdd11080239

**Published:** 2024-08-05

**Authors:** Imma Romanazzi, Antonio Di Monaco, Ilaria Bonaparte, Noemi Valenti, Alessia Surgo, Fiorella Di Guglielmo, Alba Fiorentino, Massimo Grimaldi

**Affiliations:** 1 Department of Cardiology, General Regional Hospital “F. Miulli”, Acquaviva Delle Fonti, 70021 Bari, Italy; i.romanazzi@miulli.it (I.R.); noemiv4931@gmail.com (N.V.); m.grimaldi@miulli.it (M.G.); 2 Department of Radiation Oncology, General Regional Hospital “F. Miulli”, Acquaviva Delle Fonti, 70021 Bari, Italy; ilariabonaparte@libero.it (I.B.); surgo.alessia@gmail.com (A.S.); fc.diguglielmo@miulli.it (F.D.G.); a.fiorentino@miulli.it (A.F.); 3 Department of Medicine, LUM University, 70010 Casamassima, Italy

**Keywords:** ventricular tachycardia, arrhythmias, radioablation, CardioInsight, noninvasive cardiac mapping

## Abstract

Objectives: Sustained monomorphic ventricular tachycardia (SMVT) is a life-threatening condition that is often observed in patients with structural heart disease. Catheter ablation (CA) ablation is an effective and well-established treatment for the scar-related ventricular tachycardias (VTs). Sometimes, due to patient fragility or contraindications to CA, a noninvasive procedure is required. In these cases, VT ablation with stereotactic arrhythmia radioablation (STAR) for SMVTs supported by the CardioInsight mapping system seems to be a promising and effective noninvasive approach. Methods and results: We report a case of a 55-year-old male smoker and heavy alcohol consumer who developed ischemic heart disease and frequent refractory SMVT relative to antiarrhythmic drugs. Catheter ablation was not practicable due to the presence of an apical thrombosis in the left ventricle. The CardioInsight^TM^ system (Cardioinsight Technologies Inc., Cleveland, OH, USA) was useful for noninvasively mapping the VTs, identifying two target areas on the septum and anterior wall of the left ventricle. A personalized STAR treatment plan was carefully designed, and it was delivered in a few minutes. During follow-up, a significant reduction in the arrhythmia burden was documented. Conclusions: Stereotactic arrhythmia radioablation supported by the CardioInsight system could be an alternative treatment for VTs when catheter ablation is not possible. Larger studies are needed to investigate this technique.

## 1. Introduction

Ventricular tachycardia (VT) is a life-threatening arrhythmia often occurring in structural heart disease. The prevention and termination of VT requires appropriate antiarrhythmic drugs and/or external or implantable cardioverter defibrillator (ICD) intervention (1). Three or more episodes of sustained ventricular arrhythmias within 24 h, separated by at least 5 min, requiring termination via an intervention are classified as an electrical storm (ES). Previous studies have described poor outcomes associated with ES and an up to 3-fold increased risk of mortality in patients with ES [1,2,3,4]. 

Catheter ablation (CA) for VT can eliminate or reduce recurrent arrhythmia episodes in patients affected by ES [5,6,7,8,9].

In some cases, cardiac ablation is not feasible due to particular clinical conditions, such as the presence of a double mechanical valve in the mitro-aortic site or clot formations in the left ventricle. In these clinical conditions, noninvasive therapeutic alternatives are necessary. Stereotactic arrhythmia radioablation (STAR) with precise high-dose radiation to define targets, potentially guided by previous cardiac diagnostic tools, was used to treat these patients [10,11,12]. It is crucial to collect all available pieces of information about the arrhythmogenic substrate to identify the critical isthmus of VTs in order to define an accurate treatment plan. Electrocardiographic imaging (ECGI) is a mapping technique aiming to noninvasively characterize cardiac electrical activity [13]. ECGI employs body surface electrodes combined with patient-specific computed tomography or magnetic resonance imaging-derived epicardial geometry to display the full sequence of electrical activity during a single beat over the whole heart, hence providing a panoramic map of the arrhythmia. This is achieved using an inverse method described previously [14,15] and provides sufficient resolution to identify myocardial segments with sites of earliest activation in VT, but the method is not able to identify slow conduction/fragmented potentials, as well as VT isthmuses [16]. The commercial ECGI system CardioInsight (Medtronic, Dublin, Ireland) has recently become available for clinical applications [13,16]. 

## 2. Case Presentation

**Figure 1:**
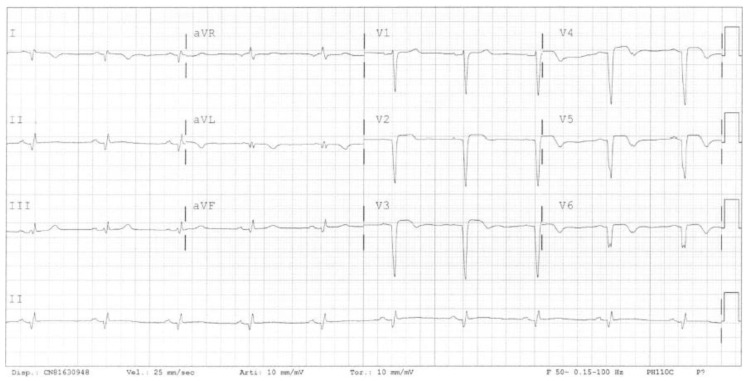
Electrocardiogram showing the sinus rhythm and signs of myocardial necrosis in anterior–lateral leads.

The clinical case focuses on a 52-year-old male smoker and heavy alcohol consumer with a family history of ischemic heart disease. His cardiological history began in 2017 when he went to the emergency room due to epigastric pain. Sustained VT was diagnosed and treated effectively with DC shocks. The ECG with the sinus rhythm showed q waves in the anterior–lateral leads, which corresponded with necrosis (Figure 1). 

Severe left ventricular dilatation, apical thrombosis, and severe impairment of LV systolic function (EF 21%) were found in the echocardiogram. Angiography was performed, and a left anterior descending artery occlusion was found. Since 68% necrosis of the left ventricle was present upon myocardial scintigraphy, no revascularization was performed. He underwent a dual chamber ICD implantation and started oral anticoagulation therapy.

**Figure 2:**
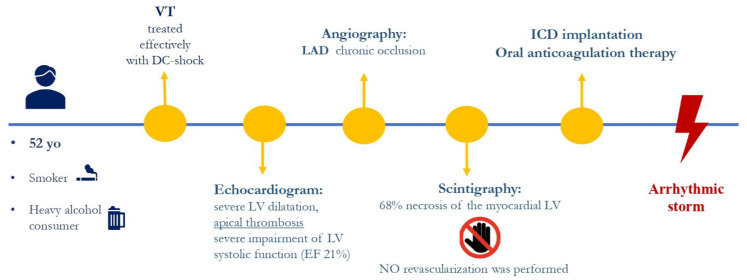
Summary of the patient’s medical history.

Despite an up-titration of antiarrhythmic therapy in the following years (maximum dosage of beta-blockers, amiodarone, and mexiletine), frequent VT recurrences were documented. The episodes were effectively treated by ICD shock (Figure 2).

**Figure 3:**
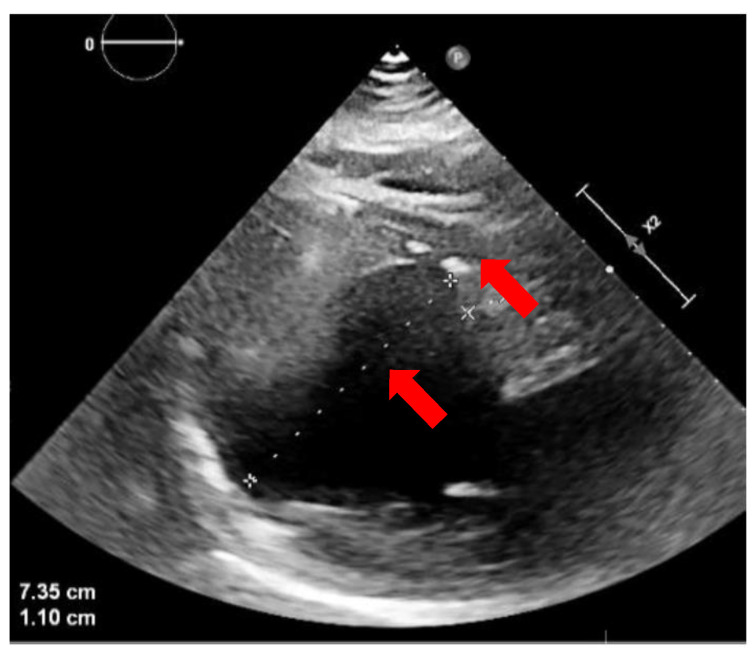
Echocardiographic image (parasternal long axis) showing a severely dilated left ventricle with apical thrombosis (red arrows).

A CA of VT was not possible due to the persistence of left ventricle apical thrombosis (Figure 3). After a careful evaluation of all possible treatments (for example, alcohol ablation or intramural needle ablation) [17], the patients underwent STAR. However, the question of which area of LV to target during STAR was a challenging problem. Cardiac magnetic resonance imaging was not performed due to the patient’s claustrophobia and refusal of general anesthesia. The presence of the defibrillator with two catheters would also have reduced the quality of the images.

A noninvasive mapping of VTs using CardioInsight was performed. The 252-electrode vest was applied to the patient’s torso and connected to the system. A CT scan allowed the definition of cardiac anatomy and the position of each electrode on the torso. LV geometry was then reconstructed to obtain a three-dimensional mesh. This model serves as the projection of unipolar signals represented by virtual nodes of the epicardial surface. The collected signals are post-processed using mathematical reconstruction algorithms to create different maps, including activation maps, voltage maps, isopotential maps, and phase maps [13].

**Figure 4:**
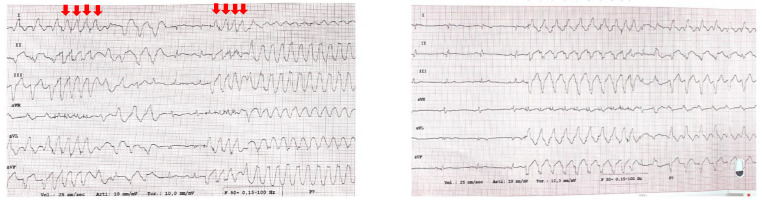
Ventricular tachycardias induced by programmed ventricular stimulation using ICD. The red arrows correspond to the stimulated beats.

**Figure 5:**
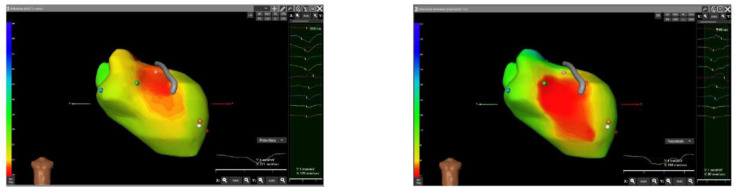
Noninvasive mapping of ventricular tachycardia (VT) using the CardioInsight system. Two VTs were induced and mapped. On the left, the system identified the anterior wall as the site of earliest activation during the VT1 (red area). On the right, the system identified the middle septum as the site of earliest activation during VT2 (red area). The right anterior oblique (RAO) projection is shown for all images.

A programmed ventricular stimulation was performed using the ICD, and two VTs were induced (Figure 4). The CardioInsight system mapped the VTs noninvasively and studied the areas of maximum slowing. The system identified the middle septum and anterior wall of the left ventricle as target areas (Figure 5).

**Figure 6:**
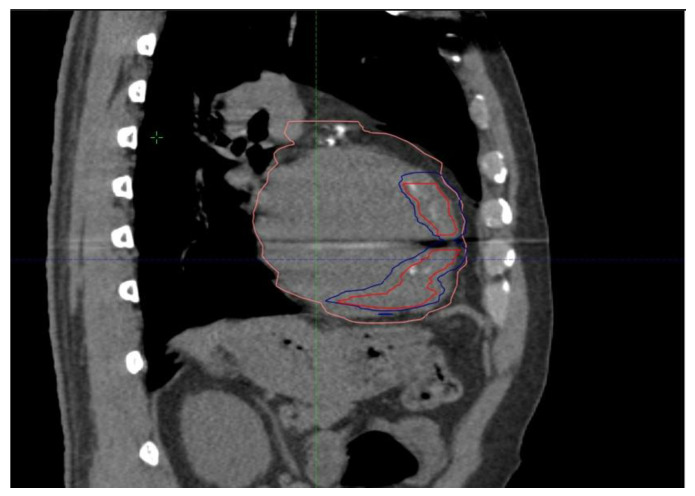
Treatment plan for STAR. The area outlined by the red line corresponds to the Clinical target volume (CTV). The area outlined by the blue line corresponds to the Planning target volume (PTV). The area outlined by the pink area corresponds to the heart volume.

Thanks to cooperative work with radiation oncologists and medical physicists, a personalized treatment plan was created to deliver an effective high dose of radiation to the target areas, minimizing exposure to nearby organs at risk [12]. The clinical target volume (CTV) was identified in accordance with radiation oncology and cardiology, considering the scar area based on anatomical CT imaging. Based on the 4D-CT acquisition, an internal target volume (ITV) was added to CTVs in order to compensate for respiratory motion. Finally, the planning target volume (PTV) was defined by adding 0–2 mm to the ITV (Figure 6).

The ICD lead was completely excluded from the treatment plan. In particular, the patient was immobilized in the supine position using a vac-lock bag, and three CTs were performed: basic free-breathing CT for dose calculations, four-dimensional CT for moving evaluation, and CT with contrast for anatomical accuracy. Several organs at risk were contoured, paying more attention to the esophagus and main bronchus, for which a planning risk volume was built. 

In February 2023, STAR was performed via free breathing with a planning target volume prescription total dose of 25 Gy/1 fraction. For OARs, all dose constraints were respected [18] (esophagus max dose of 5.5 Gy, aorta max dose of 1.6 Gy, superior vena cava max dose of 5.1 Gy, and inferior vena cava max dose of 0.3 Gy). The heart–PTV mean dose was 4.7 Gy. 

**Figure 7:**
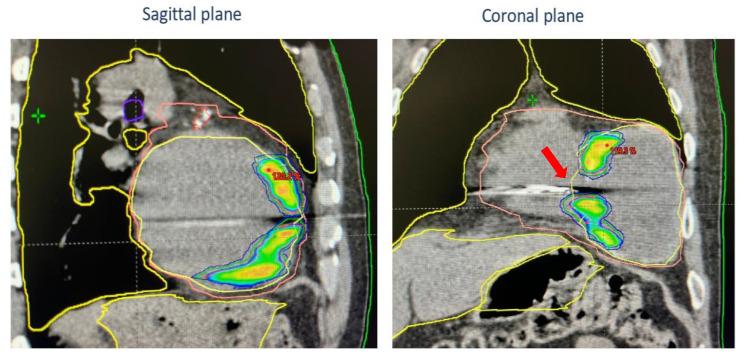
Dose distribution and treatment plan shown in sagittal CT images (**left**) and coronal plan (**right**). The areas outlined by the yellow, violet and red lines correspond to the organs at risk (OARs). The area outlined by the pink area corresponds to the heart volume. Colored areas correspond to isodose level of RTplan. Notice that the ICD lead was excluded from treatment (red arrow).

The treatment was generated, optimized, and delivered via TrueBeam^TM^ (Varian Medical Systems, Palo Alto, CA, USA). Image-guided radiotherapy (IGRT) involves the use of cone-beam CT to capture detailed images of a patient’s anatomy before the treatment session, allowing for the precise targeting of the area to be treated and minimizing setup errors. In addition, surface-guided radiotherapy with Align-RT is used to monitor and adjust patient positioning in real time during treatment. These advanced techniques enhanced the overall precision and safety of the STAR process (Figure 7).

The treatment lasted 3 min, and no acute complications were documented. The patient was quickly discharged in good clinical condition. At the post-STAR echocardiography, the LV systolic function remained unchanged. The patient was discharged with mexiletine therapy, and amiodarone was discontinued.

After three months of clinical well-being observation, the patient reported a new single episode of VT treated with ICD shock in June 2023 (12-lead ECG showing VT was not available; cycle length of VT was different from that of previously treated VT). No other VT episodes or side effects due to STAR were documented after a one-year follow-up.

## 3. Discussion

The introduction of STAR offers a new treatment option for patients with recurrent episodes of VT when cardiac ablation is not feasible [10,11,12]. There is still debate regarding whether STAR has a predominant effect in the early or late period after the procedure. The majority of studies claimed that the time course of the decline in VT attacks after STAR was acute [19,20]; in contrast, Neuwirth et al. [21] and, more recently, Kautzner et al. [22] indicated that STAR had predominantly delayed effects after 6–12 months due to progression of fibrosis. The effects of radioablation seemed to occur immediately after the procedure, implying that the reduction in VT burden cannot be attributed solely to fibrosis but previous studies reported that myocardial inflammation and structural changes can be induced within a month after STAR [23,24,25]

STAR can be used for immediate antiarrhythmic palliation in critically ill patients with otherwise untreatable refractory VT and ES. The CardioInsight system is a very useful tool for optimizing the effectiveness of STAR by generating accurate activation maps and optimizing the radiotherapy treatment plan. Prior studies [16] have shown that the distance between the VT isthmus performed using electroanatomical mapping (sites of entrainment, pace-mapping, and termination using CARTO, Biosense-Webster) and sites of earliest activation in ECG imaging (CardioInsight, Medtronic) was 22.6 mm (median). This simultaneous assessment demonstrates that CardioInsight localizes VT circuits with sufficient accuracy to provide a region of interest for ablation. The resolution, however, is not sufficient for guiding discrete radiofrequency lesion delivery via CA without the concomitant use of an electroanatomical mapping system, but it may be sufficient for segmental ablation with radiotherapy [16].

In fact, in contrast to traditional tumor RT planning, the arrhythmogenic substrate cannot be directly visualized on the 4D-CT scan, making target definition and delineation more complex. Therefore, the delineation process is based on a collaborative and subjective synthesis of many variables. To date, no clinically validated methods or commercial products are available for transporting the identified arrhythmogenic targets from the electroanatomical mapping systems to the radiation oncology treatment planning systems. Imaging techniques such as MRI and CT scans are often used, and they are able to identify, in areas of dense fibrosis, corridors and areas of tissue that are potentially capable of generating reentry circuits. These techniques, when used as the only method to establish the target volume, risk the resultant treatment of areas not responsible for the arrhythmia. The possibility of visualizing early activation during VT using the CardioInsight system is a very useful method for improving STAR. 

## 4. Conclusions

The introduction of STAR guided by the CardioInsight system offers a new treatment option for patients with recurrent episodes of VT when cardiac ablation is not feasible. Further studies are necessary to demonstrate the safety and effectiveness of this technique.

## Data Availability

Data is contained within the article.
